# Cognitive and Memory Functions in Plant Immunity

**DOI:** 10.3390/vaccines8030541

**Published:** 2020-09-17

**Authors:** Hidetaka Yakura

**Affiliations:** Institute for Science and Human Existence, Tokyo 163-8001, Japan; she.yakura@gmail.com

**Keywords:** autoimmunity, cognition, metaphysicalization, plant immunity, transgenerational memory

## Abstract

From the time of Thucydides in the 5th century BC, it has been known that specific recognition of pathogens and memory formation are critical components of immune functions. In contrast to the immune system of jawed vertebrates, such as humans and mice, plants lack a circulatory system with mobile immune cells and a repertoire of clonally distributed antigen receptors with almost unlimited specificities. However, without these systems and mechanisms, plants can live and survive in the same hostile environment faced by other organisms. In fact, they achieve specific pathogen recognition and elimination, with limited self-reactivity, and generate immunological memory, sometimes with transgenerational characteristics. Thus, the plant immune system satisfies minimal conditions for constituting an immune system, namely, the recognition of signals in the milieu, integration of that information, subsequent efficient reaction based on the integrated information, and memorization of the experience. In the previous report, this set of elements was proposed as an example of minimal cognitive functions. In this essay, I will first review current understanding of plant immunity and then discuss the unique features of cognitive activities, including recognition of signals from external as well as internal environments, autoimmunity, and memory formation. In doing so, I hope to reach a deeper understanding of the significance of immunity omnipresent in the realm of living organisms.

## 1. Introduction

The general picture of how immunity operates has been largely established on the basis of the findings in jawed vertebrates (gnathostomes), specifically mice and humans [1,2]. In addition to non-specific innate defense mechanisms, these vertebrates have a circulatory system with mobile immune cells and a repertoire of clonally distributed antigen receptors with an almost unlimited number of specificities, generated by somatic recombination and mutation of T and B cell receptor genes. More recently, it has been shown that jawless vertebrates (agnathans) also have an adaptive immune system that is based on recombinatorial assembly of different types of genetic units to generate a highly diverse repertoire of lymphocytes, called a variable lymphocyte receptor (VLR) [3]. In contrast, plants lack mobile immune cells with a highly specific recognition system, and yet live and survive in the same harsh environment. In fact, plants can perform specific recognition of pathogens, induce self-tolerant immune responses, and generate a lifelong or even transgenerational memory of the encounters with pathogens [4,5,6,7,8,9]. In the case of jawed vertebrates, the initial recognition of an antigen by the corresponding receptor leads to the specific activation and differentiation of lymphocytes and the generation of long-lived memory cells with the same antigen-binding specificity such that a second encounter with the corresponding antigen results in faster and more vigorous immune responses. How do plants manage to achieve recognition of the virtually unlimited number of pathogens and to generate immunological memory? How should the characteristics of plant immunity be positioned within the phenomenon of immunity in the living world? Moreover, how are the philosophical significance and essential feature of immunity defined? To answer these questions, I will apply what I call the “metaphysicalization of science” [10,11]. This method consists of two steps: (1) extraction of minimal and essential components in scientific findings and (2) meditative and logical reflection and reasoning on the extracted facts. In the present case, the first step is to investigate what is known about the structure and the mechanism by which plants maintain organismal integrity by coping with disturbing signals from external as well as internal environments, and to extract minimal and essential features of what constitutes plant immunity. The second step concerns a philosophical and more general reflection on the extracted features to reach a deeper understanding of plant immunity and immunity in general.

## 2. Immunological Machinery in Plants

Microorganisms, such as viruses, bacteria, fungi, and oomycetes, must enter the plant interior to be pathogenic. This can be accomplished by directly penetrating the leaf or root surface or by invading wounds or physiological openings, such as gas pores (stomata) and water pores (hydathodes). The first line of defense against phytopathogens is the plant cell wall, not only as a physical barrier against biotic and abiotic stresses but also as a dynamic regulator of host defense [12,13,14]. At the site of penetration, the polysaccharide callose is produced and deposited to reinforce host defense. However, plant pathogens use a variety of strategies to attack plants. For example, plant pathogenic bacteria deliver effectors (virulence factors) into host cells by the type III secretion system. Fungi and oomycetes invaginate feeding organelles (haustoria) into host cells. Nematodes and aphids feed salivary proteins by inserting a stylet directly into a plant cell. The specific line of defense in plants is mediated by innate immunity that functions against bacteria, fungi, and oomycetes via two separate classes of receptors. In this essay, the immunity executed by RNA silencing mechanisms against viruses and transposable DNA elements [15,16] will not be covered.

### 2.1. Structure of Plant Innate Immunity

The first class of innate immunity operating at the plant cell surface is mediated by transmembrane pattern recognition receptors (PRRs) that detect evolutionarily conserved molecular patterns of microorganisms, called pathogen-associated molecular patterns (PAMPs) or microbe-associated molecular patterns (MAMPs) [17,18]. In the following discussion, the term MAMPs is used throughout. Given that MAMPs are not present in the host under physiological conditions, they are recognized as foreign by the host. MAMPs include, among others, lipopolysaccharide (LPS: a major component of the outer membrane of Gram-negative bacteria), peptidoglycans (polymers consisting of sugars and amino acids that form an outside layer of the bacterial plasma membrane), flagellin (the protein subunit of the bacterial flagellum), chitin (a component of the cell walls of fungi, among others), and ergosterol (fungal-specific glycosylated proteins) [19]. In the perception of MAMPs by PRRs, the decomposition and release of MAMPs, for example, peptidoglycans, by a host-derived lysozyme-like hydrolase may aid PRR-mediated activation [20]. All known plant PRRs are receptor-like serine/threonine kinases or receptor-like proteins. The former consists of an extracellular domain with leucine-rich repeats or lysine motifs, a transmembrane domain, and an intracellular serine/threonine kinase domain. The latter receptor-like proteins have an extracellular domain but not a kinase domain [21]. The interaction between MAMPs and PRRs leads to a series of biochemical and cellular changes, including MAP kinase activation, transcriptional induction of pathogen-responsive genes, and production of reactive oxygen species to prevent microbial proliferation [22]. This process is called PRR-triggered immunity (PTI). In most cases, infection is prevented by this first tier of defense. However, some adapted pathogens secrete multiple effector molecules inside host cells to evade or suppress PTI, enhancing disease susceptibility [18,23].

At this stage, the second class of innate immunity operating inside the cell is initiated. This process is triggered by nucleotide-binding oligomerization domain (NOD)-like receptors (NLRs) that directly or indirectly detect highly polymorphic strain-specific pathogen effectors in the cell interior. Thus, the second arm of innate immunity is termed effector-triggered immunity (ETI). The NLRs were originally defined as plant resistance (R) proteins that confer resistance to bacteria, viruses, fungi, oomycetes, nematodes, and even insects [24,25,26]. ETI is often associated with the hypersensitive response (HR), a type of programmed cell death of the infected cells and the production of antimicrobial enzymes, thereby preventing the invading pathogen from further spread. A local HR then induces immune responses throughout the whole plant, which is called systemic acquired resistance (SAR). This phenomenon is characterized by a broad-spectrum resistance, as evidenced by the original report by Frank Ross, showing that the tobacco mosaic virus (TMV) can induce protection not only from TMV but also other pathogens [27]. Importantly, this report also demonstrated that the broad-spectrum resistance lasts for 20 days. From the coevolutionary perspectives of host–microbe interactions, it is possible to see the relationship between PTI and ETI in the following sequences [4]. After PTI was successful, pathogens acquired the strategy to inject effector proteins into the plant cell interior to suppress PTI. Some plants, in turn, evolved to produce R proteins to check for the presence of effector proteins.

### 2.2. Specific Recognition in Plant Immunity

Historically, the first report on Mendelian inheritance of disease resistance in plants appeared in 1907 [28], and the seminal experiments by Harold Henry Flor showed the genetic basis of resistance and recognition specificity in flax to the rust fungus *Melampsora lini* [29,30,31]. From these observations, Flor proposed a conceptual framework, called the “gene-for-gene” hypothesis of disease resistance [32,33]. The hypothesis states that resistance in the host is controlled by the matching of two genes: one is a single *R* gene in plants and the other is the avirulence (*avr*) gene of pathogens that is responsible for their ability to cause disease. R protein as a receptor directly binds the effector protein or *avr* gene product as a ligand. Plants that produce a specific R protein are resistant to a pathogen producing the corresponding *avr* gene product. There are indeed reports showing that R proteins directly interact with Avr proteins of pathogens [34,35,36], but extensive attempts to detect such direct interactions between receptor and effector have been unsuccessful. Furthermore, the hypothesis predicts that there should be enough *R* genes to correspond to the number of potential pathogens, but the repertoire of *R* genes is fairly limited—only approximately 150 *R* genes in *Arabidopsis thaliana* [37] and 600 in rice *Oryza sativa* [38], for example. Thus, the direct binding or receptor–ligand model of R and Avr proteins is either untenable or explains only a part of the complete reality.

How then can plants with the limited number of R proteins manage to fight against a vast array of pathogens? The indirect binding or guard hypothesis [18,24] was proposed to reconcile this dilemma. This hypothesis posits that R proteins do not directly bind pathogen proteins but bind or “guard” host proteins perturbed by pathogen effectors. Thus, such a host protein is named “guardee”. The basic tenets of this hypothesis are (1) an effector acting as a virulence factor has a target(s) in the host, (2) the effector contributes to pathogenesis in susceptible hosts by manipulating or modifying host target(s), and (3) a host target perturbed by pathogen effectors generates a “pathogen-induced modified-self” molecular pattern, which in turn activates the corresponding R receptor protein. Thus, R proteins indirectly perceive the intrusion of pathogens by monitoring changes in self-proteins and initiate a defense response. This hypothesis predicts the following: (1) multiple effectors could independently evolve to alter the same host target, (2) this could drive the evolution of more than one R protein associated with a target of multiple effectors, and (3) these R proteins would be activated by recognizing different modified-self patterns on the same target induced by the multiple effectors [18].

These predictions have been experimentally confirmed. One of the best-studied host targets of ETI is the resistance to *Pseudomonas syringae pv. maculicola 1* (RPM1)-interacting protein 4 (RIN4) of *Arabidopsis thaliana*, a 211-amino-acid, acylated, and plasma membrane-associated protein. In unchallenged states, RIN4 binds the R proteins RPM1 and resistance to *Pseudomonas syringae* 2 (RPS2), maintaining R proteins in an inactive state. Upon infection with pathogens, such as *Pseudomonas syringae*, the effector molecules, AvrRpm1 and AvrB, are injected into the host cell, resulting in threonine phosphorylation of RIN4 by RPM1-induced protein kinase (RIPK) [39,40]. The phosphorylated RIN4 in turn binds and activates host R protein RPM1, possibly through its intramolecular conformational changes. In this process, RPM1 perceives RIN4 phosphorylation as a change in self component and is activated, triggering downstream signaling events of ETI [41,42]. In the case of the third effector AvrRpt2 (a cysteine protease of *Pseudomonas syringae*), RIN4 is proteolytically cleaved, which is sensed by R protein RPS2, eventually leading to ETI [40,43,44]. Further experiments demonstrated that RIN4 is not the only target for AvrRpm1 or AvrRpt2 and that AvrRpt2 can cleave *Arabidopsis* proteins other than RIN4, suggesting that a particular effector modulates many self-proteins. These results indicate the plant immune system does not directly recognize nonself molecules in most cases but monitors pathogen effector-induced alterations of self-molecules. In this way, plants can deal with a greater number of pathogens that utilize effectors acting on the same host targets. This unexpected strategy developed by plants addresses how a limited repertoire of R proteins maximizes the plants’ capacity to defend against a large array of pathogens.

For vertebrates, Polly Matzinger proposed the danger theory to explain the initiation of immune responses [45]. This theory posits that some PRRs in the antigen-presenting cells do not directly recognize pathogens or non-self but danger-associated molecular patterns (DAMPs), which represent host molecules or tissues damaged or stressed by infections or other causes. Thus, it argues that the initiation process is not based on self–nonself discrimination, because the critical criterion is whether a particular element is harmful to the host, rather than whether that is self or not. Conceptually speaking, the danger theory for the vertebrate immunity is similar to the guard theory proposed for plants. In the danger theory, however, the molecular nature of “danger” has not been well characterized such that it is hard to predict whether an immune response occurs under a specific condition. Matzinger later proposed a new thesis from the evolutionary point of view that one of the criteria of danger is the hydrophobicity of the molecules [46]. Given that life begins in water and the hydrophobic portion of a molecule is entirely internal, the hydrophobic portion, if exposed, could become a danger to the milieu of the cell. However, there is still much to be done to clarify the nature of danger. In contrast, the nature of self-proteins and their modifications to be monitored in plants is better characterized in molecular terms.

### 2.3. Local Hypersensitive Response and Systemic Acquired Resistance

One characteristic of the plant immune system is that, in contrast to animals, there are no mobile immune cells and antibodies. A question naturally arises as to how plants manage to control pathogenic attacks systemically [7]. There must be equally effective immune mechanisms that guarantee successful protection from pathogens in plants. Before investigating this question, we first examine local events in greater detail. As indicated above, ETI is triggered by the interaction between a pathogen effector and a host R protein. A specific local event is an HR that assumes a form of programmed cell death, which is actively induced by a tightly regulated sequence of biochemical events [47]. Whereas in animals, NLRs can trigger cell apoptosis by the action of caspases [48], there are no similar caspases in plants. Thus, plants have to utilize alternative strategies for R protein-mediated programmed cell death. In the response of *Arabidopsis thaliana* to the pathogenic bacteria *Pseudomonas syringae* pv. *tomato*, proteasome β-subunit A1 (PBA1) serves in a caspase-3-like capacity. This role includes involvement in the fusion of the membranes of a large central vacuole with the plasma membrane, which leads to the discharge of vacuolar antibacterial proteins to the outside of the cells, contributing to both antibacterial and cell death-inducing activity [49]. It has also been reported that programmed cell death in *Arabidopsis* is controlled by two type I metacaspases, AtMC1 and AtMC2, the first of which is a positive regulator of cell death, and the second, a negative regulator [50]. HR is thus effective against a variety of pathogens, including viruses, bacteria, fungi, and nematodes. In certain experimental conditions, there is a dissociation between cell death and R protein-mediated pathogen resistance, raising the question of whether cell death is the cause or one of the consequences of resistance [51,52].

After local HR, SAR is induced throughout the plant. This phenomenon was first reported by Frank Ross more than a half-century ago [27]. What Ross found was that tobacco plants locally infected with TMV are systemically protected not only against this virus but also against other viruses. Questions then arise as to the mechanism whereby distant uninfected parts of a plant are primed to activate enhanced defense responses upon secondary pathogen attack and to the nature of “immune signals” that are supposed to be disseminated from the site of infection throughout the organism. In plants, although there is no circulatory system like that found in animals, the vascular tissue, called phloem, is present. After SAR is triggered, the hormone salicylic acid increases in the phloem. The elimination of salicylic acid by expressing the gene for salicylate hydroxylase that converts salicylic acid to catechol results in impairment of the ability to develop SAR [53,54], suggesting that salicylic acid is essential to the development of SAR. However, salicylic acid is not responsible for the process of signal generation per se but seems to be required for later stages of SAR. Other candidate immune signals include methylsalicylic acid [55], jasmonic acid [56], glycerol-3-phosphate [57], and azelaic acid [58], among others. If mobile immune signals transport information about primary pathogens to distal uninfected parts of a plant, it is probable that hormonal cross-talks take place between these mobile signals to counteract most effectively a wide range of pathogens.

### 2.4. Autoimmunity in Plants

As discussed above, the plant immune system principally functions by recognizing non-self or self-proteins modified by pathogen effectors. NLR receptor proteins involved in this function are expressed on all plant cells and each cell can initiate an effective immune response, conferring disease resistance. These immune receptors are tightly regulated by the negative regulators such that they remain inactive in the absence of pathogens and become activated only upon encounters with pathogens. However, an accident can occur when two different immune systems meet in the offspring of crosses, as intraspecific and interspecific crosses sometimes result in a mismatch between NLRs and their guardee proteins. NLR proteins from one parent recognize the effector targets or guardee molecules from another parent as the modified self-antigen, thus leading to autoactivation of the NLRs in the absence of pathogens, called hybrid necrosis [59]. Inappropriate activation of the respective NLRs results in dwarfism; macroscopic lesion formation; and, in severe cases, the death of all F_1_ hybrids. The *DANGEROUS MIX 1* (*DM1*) and *DM2d* alleles in *Arabidopsis*, when combined, trigger tissue necrosis and seedling lethality [59,60]. Two unlinked NLR alleles recombine to produce heteromeric association that causes aberrant immune activation. The DM proteins have been confirmed to function as the hyper-modulated NLR complex [61]. Thus, imbalanced NLR activity can trigger autoimmunity in hybrid plants.

A study of hybrid necrosis in lettuce (*Lactuca sativa*) showed that one of the loci is a gene for an orthologue of a target protein RIN4 in *Arabidopsis thaliana* [62]. Another study of *Arabidopsis* revealed that hybrid necrosis arises from incompatible interactions between the *RPP1* (recognition of *Peronospora parasitica* 1) cluster of *NLR* genes and allelic variations in the gene encoding the leucine-rich receptor-like kinase, STRUBBELIG receptor family 3 (SRF3) [63]. This result suggests that NLR proteins encoded by *RPP1* genes might be involved in the monitoring of pathogen effector-induced changes in SRF3 kinase. Mutational studies of *NLR* genes demonstrated that enhanced activity or overexpression of NLR proteins leads to autoimmunity [64,65,66], suggesting that there must be mechanisms whereby the activity and the level of NLR proteins are strictly controlled under normal conditions. Mutation of genes involved in the transcriptional regulation of *NLR* genes, such as suppressor of effector-triggered immunity (*SRFR1*), showed that NLR protein accumulation results in autoimmunity [67,68]. Similarly, if genes involved in the systems of protein degradation that control the level of NLR proteins are functionally eliminated, NLR proteins indeed increase, inducing autoimmunity [69]. The fact that this phenotype can be reversed by eliminating one of the *NLR* genes indicates that this is due to excessive accumulation of NLR proteins. Thus, tight regulation of the activity and the expression of NLR proteins is required to avoid autoimmunity.

### 2.5. Immunological Memory in Plants

Given that memory formation is one of the hallmarks of immunity, how does the immune system of plants remember the encounters with pathogens and react faster and more vigorously to a future encounter with the same pathogens? It has been known for some time that plants are induced to a primed state of enhanced defense. This process, called defense priming or trained immunity, was first proposed in plants by Kenneth Chester in 1933 [70]. Earlier studies demonstrated that various plants could protect against a variety of diseases, suggesting that memory is not restricted to an original pathogen but has a characteristic of broad-spectrum resistance [71]. Mechanistic aspects of this phenomenon have been examined only in recent years. Defense priming is accompanied by SAR and is activated by immune signals, such as salicylic, jasmonic, and azelaic acids. Signaling mechanisms have also been partially elucidated. For example, in *Arabidopsis thaliana*, defense priming even with local infection is correlated with the systemic accumulation at the level of mRNA and protein of the mitogen-activated protein kinases MPK3 and MPK6, and the elimination of the function of these two enzymes attenuates priming [72]. Furthermore, the nonexpressor of pathogenesis-related genes 1 (NPR1), a transcription cofactor that activates immune-related genes and genes for transcription factors, is also involved in this process [73,74]. Thus, immune signals, such as MPK3, MPK6, and NPR1, are critical to long-lasting memory for enhanced secondary responses against pathogens.

A recent study demonstrated that the immune memory of SAR activated by inoculation with *Pseudomonas syringae* pv. *tomato* in *Arabidopsis* is passed on to the following generation [75]. The progeny exhibited enhanced resistance not only to *Pseudomonas syringae* pv. *tomato* but also to the unrelated oomycete *Hyaloperonospora arabidopsidis*, an obligate parasite and the causal agent of the downy mildew in *Arabidopsis*. Furthermore, the progeny from the *npr1* gene mutant failed to develop transgenerational memory, indicating an essential role for NPR1 in transgenerational SAR. Given that this transgenerational SAR was sustained over a stress-free generation, the authors proposed that an epigenetic mechanism is involved in this phenomenon. Indeed, it was discovered to be associated with changes in the methylation and acetylation state of histones of various *npr1*-regulated or SAR-associated genes. The authors also showed that DNA methylation status is linked to transgenerational transmission of SAR memory. The reduced DNA methylation in the triple mutant of *drm1* (domains rearranged methyltransferase (1), *drm2* (domains rearranged methyltransferase (2), and *cmt3* (cytosine methyltransferase (3) mimics transgenerational priming of SAR-dependent defenses [75], suggesting that hypomethylated DNA may mediate the transmission of this defense priming.

## 3. Philosophical Reflections on Plant Immunity and Immunity in General

In the previous sections, that which science has revealed regarding the structural and mechanistic characteristics of plant immunity has been reviewed. For those learning this information for the first time, it may be surprising that plants possess such exquisite structures and mechanisms and perform such logical molecular interactions to maintain their integrity. Although the structural features of the plant immune system are quite different from those of other organisms, the functional elements and the logic of cause and effect are surprisingly similar. In this section, the following three major problems surrounding plant immunity will be addressed, namely, recognition of pathogens, autoimmunity, and memory formation. Furthermore, the philosophical significance of immunity, in general, will be discussed. In formal scientific experimentation, the final philosophical step is omitted or even prohibited, but the “metaphysicalization” is critical for a deeper comprehension of nature and should be included in scientific activities. The point to be stressed here is that the activity of philosophical contemplations with logical reasoning is necessarily based on scientific facts but is not through a priori meditation. If the term directly related to “metaphysics” sounds awkward or inappropriate, this framework can be called the “ethics of knowledge”, in the sense that it is desirable and valuable to incorporate this process in the final analysis of scientific discoveries for a deeper understanding of nature or any other phenomena.

### 3.1. Search for the Essential Feature of Immunity

In a previous report [76], the essential feature of immunity was tentatively defined as the minimal requirements for its functional performance but not its structural organizations. Because the wealth of information on immunity has been accumulated in humans and mice, anthropocentrism is one possible outcome. By concentrating on the structural elements, it is easy to miss that which can be called immunity or an immune system in other organisms. Recent advances in this field have revealed that almost all organisms, from bacteria to humans, have a function called immunity. Thus, it would be difficult to grasp the whole picture of immunity in highly evolved organisms and to extract minimal elements that constitute immunity, for example, from humans, because their functions are diversified, multifactorial, highly interactive, and nuanced. Thus, my analysis was focused on the most primitive bacterial and archaeal immune system—the clustered regularly interspaced palindromic repeats (CRISPR)-CRISPR associated protein (Cas) system [77,78,79]. As a result, four functional elements were identified as an essential feature of immunity—namely, a perception of elements in a milieu, integration of perceived information, reaction according to integrated information, and memory of that experience. These elements were proposed as an example of minimal cognition [76]. Thus, immunity is coextensive with the concept of cognition in the sense given above. To reiterate, these are functional components without any structural restrictions. Accordingly, it is impossible to identify whether a specific structure is indeed an immune system or not. In a sense, immunity is a metaphysical entity, and to ask about the essence of immunity is a metaphysical act, such that you must understand it only by abstract thinking.

In humans, there are two types of memories, immunological and mental, whereas, in bacteria and archaea, immunological memory is maintained by the CRISPR-Cas system. However, these organisms have no apparent nervous system and, therefore, no mental memory. Given this fact and the assumption that the presence of adaptive and cognitive functions is indispensable for an organism’s existence and survival, there must be some neural-like mechanisms in any organism. I thus proposed that signal-initiated cognitive mechanisms exerted by the CRISPR-Cas system serve a neural-like function in bacteria and archaea. If this premise is accepted, the immune system is a more universal and fundamental cognitive system in living beings than the nervous system [76]. On the basis of this understanding, I have analyzed metaphysical implications of immunity by applying philosophical concepts, because the neural-like functions embedded in immunity seemed to overlap with the Spinozist concept of *conatus* [80]. This concept implies the inner force towards the preservation of all existence and constitutes the essence of any being (Proposition VI and VII of Part III). Furthermore, *conatus* has a mental element with the normative activity controlling biological polarity, such that the effort of maintaining balance is good but disturbing balance is bad (Proposition XXXIX of Part IV).

The *conatus* is an umbrella concept embracing inanimate things and living organisms. In the case of living organisms, the *conatus* applied only to the mind is called “will”, and the *conatus* applied to both the mind and body is named “appetite” (Proposition IX, Scholium of Part III). There is also a faculty called “desire”, which represents a condition in which human beings become conscious of “appetite” (Proposition IX, Scholium of Part III). These two mental states of *conatus* are reminiscent of the present-day definition of two levels of consciousness. One consists of the qualitative or experiential awareness of your environment and your body, and the other is recognition of that awareness. Ned Block named the former phenomenal consciousness and the latter access consciousness [81]. Later, Gerald Edelman similarly divided consciousness into “primary or sensory” consciousness and “secondary or higher-order” consciousness [82]. Recent neurobiological, behavioral, and philosophical studies of living organisms suggest that the phenomenal or primary consciousness may be a shared property of almost all living beings. This concept fits very well with recent development in the field of immunology. Thus, it can be concluded that the essential feature of immunity in a metaphysical sense is a conative activity in the form of “appetite” with normative connotations. This conclusion is compatible with the developments of the notion of psychoneuroimmune or psychoneuroendocrinoimmune networks [83,84,85,86,87,88], in the sense that immunity is an expression of whole organismal functions including mental activities.

### 3.2. Recognition Function

Although plants lack mobile immune cells, all plant cells can be viewed as a type of immune cells because they initiate an immune response by recognizing pathogens and pathogen-derived virulent effectors at the cell surface and in the intracellular space, respectively. The first tier of immune defense, PTI, is composed of cell surface receptors, PRRs, and the evolutionarily conserved molecular patterns of pathogens that PRRs recognize, namely, MAMPs. The second tier of immune defense, ETI, concerns the recognition of pathogen-derived effector molecules either directly or indirectly. In most cases, virulent effector factors modify selected target molecules in the host, and ETI indirectly detects pathogen attacks by monitoring molecular changes in self-targets induced by effectors. In other words, PTI deals with molecules from external organisms at the cell surface, whereas ETI intracellularly oversees changes of self-components induced by externally derived molecules. The strategy of plants implies that, in addition to coping with the exterior, the immune system must inherently face the self and understand its status to maintain the integrity of the organism.

In jawed vertebrates, both microbe-derived MAMPs and danger signals DAMPs originating from the self are recognized by antigen-presenting cells. This process is finished in the first phase of immune responses, the innate arm. The second phase concerns recognition by T and B lymphocytes with clonally distributed antigen receptors, the hallmark of acquired immunity. Without this sophisticated process of generation of lymphocytes with almost unlimited specificities, plants can survive. One of the reasons is the systemic nature of plant immunity that substitutes the lack of lymphocyte-like cells and antibodies. The location of each cell is fixed, but upon HR, the messages in the form of soluble factors, such as salicylic acid, jasmonic acid, and azelaic acid, are delivered throughout the plant in the phloem in SAR. Another reason is that with limited resources to distinguish external invaders directly, the plant evolves the system of utilizing self-components as monitors for invasion.

This self-referential nature of the plant immune system is reminiscent of not only of Polly Matzinger’s danger theory [45] but also Irun Cohen’s “immunological homunculus” [89,90]. According to Cohen’s definition, the adaptive and innate repertoires of the healthy immune system include receptors that recognize a defined set of body molecules. These self-recognizing receptors bind to a functional immune image of key body molecules. The immunological homunculus reads the immunogenic state of the body or can be viewed as the immune system’s picture of the self [91]. This characteristic may be universal in the immune system because autoimmunity is present even in bacteria, as briefly touched upon in the next section.

### 3.3. Autoimmunity

Autoimmunity is a condition wherein an immune machinery interacts or, in some cases, destructs the self-components. At the turn of the 20th century, Paul Ehrlich performed a series of experiments on the assumption that self-produced hemolytic antibodies may contribute to the physiological destruction of worn-out erythrocytes. What he found was that if goats were injected with red blood cells from other species or even from other goats, the recipients produced antibodies that lysed the erythrocytes, whereas the same goat never produced antibodies against its own red blood cells. Ehrlich thought that, in principle, antibodies against self-components are not produced and that this regulatory mechanism is “of the highest importance for the individual.” He coined the dictum *horror autotoxicus*, meaning the “fear of self-toxicity or self-destruction”, to indicate that the immune system avoids autoaggression because that will exert disastrous effects on the organism [92]. Unfortunately, for the half-century that followed, that view had been transformed into the belief that autoimmunity never exists.

Since the unveiling of our immune system’s ability to produce antibodies with an almost unlimited number of specificities and its genetic mechanisms, autoimmunity has been known to be an inevitable phenomenon. Indeed, when the regulatory mechanisms to avoid autoimmunity, including clonal deletion, clonal anergy, and dysregulation of regulatory T cells, go awry, autoimmunity occurs. Furthermore, the production of autoantibodies and the presence of autoreactive T cells are observed in physiological conditions. As Irun Cohen speculated [89,90], autoimmunity is not something to be avoided but necessary for the immunological homunculus to monitor the immunogenic state of the body. Self-reactivity serves as a set of biomarkers that help the immune system initiate and regulate the inflammatory processes that maintain the body [93]. As discussed above, this view of immunity overlaps with a plant’s surveillance system in which ETI monitors changes in self-components induced by pathogen-derived effectors. In plants, autoimmunity, as well as its avoidance mechanisms, are also present. Autoimmunity is the apparent fate of organisms equipped with an immune system.

Autoimmunity is indeed present from the first appearance of life on earth. In the bacterial immune system CRISPR-Cas, 0.4% of all spacers are directed against self-genes, and approximately 18% of the organisms with CRISPR-Cas have at least one self-targeting spacer [94]. An immune reaction occurs whenever a sequence of the invasive DNA matches with that of the spacers placed between CRISPR repeats. However, it is surprising but logical that a mechanism of tolerance or avoidance of self-reactivity is already developed in bacteria. According to one study, mismatches between CRISPR RNA (crRNA) and the target at specific positions outside of the spacer sequence guarantee interference of invading DNA, and extended pairing between crRNA and CRISPR DNA repeats prevents autoimmunity [95]. This differential complementarity outside of the spacer sequence determines self–nonself discrimination and the prevention of autoreactivity. It is not yet known whether the incorporation of self-DNA is a result of mere accident or is caused by the recognition of self-DNA in viruses or plasmids as foreign. In either case, it seems that bacteria adapt well to this unexpected situation by developing a new strategy to avoid autoimmunity.

### 3.4. Memory Formation

It is of particularly notable that in contrast to immunological memory in mammals, the plant immune system exhibits a broad-spectrum resistance characterized by SAR, which induces protective ability not only against an original invading pathogen but against a broad range of pathogens. This characteristic, prima facie, has beneficial effects on the survival of plants with limited immune apparatus. A similar type of memory, called trained immunity, is operative in vertebrates. According to a recent review [96], trained immunity is defined as the long-term functional reprogramming of innate immune cells, evoked by exogenous or endogenous signals, which leads to an altered response towards a second challenge. For example, the Bacillus Calmette–Guérin (BCG) vaccine was shown to protect both animals and humans against secondary infections with unrelated pathogens. Thus, its effect is non-specific but can be viewed as beneficial because of its non-specificity. However, a cautionary note here is that reprogramming of innate immunity and increased inflammatory responses may also cause harm.

Furthermore, resistance to a particular pathogen that the parents encountered during their lifetime is transmitted not only to the next generation but, in some cases, to the third or even to the fourth generations [75,97,98,99]. These results suggest that immunological memory might be a transgenerational event, first observed at the genetic level in bacteria and at the epigenetic level in plants. In vertebrates, immunological memory is executed at the cellular level, and only a few reports concerning the transgenerational transmission of memory have been reported [100,101]. Such a situation makes us wonder whether transgenerational transmission of immunological memory is only a phenomenon found in the early phase of evolution and lost thereafter or whether it somehow escapes our observations in the organisms at later phases of evolution, most probably due to a biased framework of mind or the ‘‘thought style’’ (*Denkstyl*) of Ludwik Fleck [102]. Given the recent rise of interest in epigenetics, it is worthwhile to reexamine whether, even in the vertebrate immune system, immunological memory can be transmitted to subsequent generations. With this constraint overcome, we may see the completely different unified picture of memory, which is transgenerational from bacteria to humans.

### 3.5. Immunity and Cognition

The definition of the term “cognition” may vary between authors and thus needs to be clarified for discussion. It seems that cognition generally refers to higher-ordered consciousness, roughly corresponding to the access consciousness of Ned Block and secondary consciousness of Gerald Edelman, mainly concerning human ability. Because of this perception, it is challenging to accept the cognitive capacity of other organisms or their systems. In my previous report [76] and this essay, cognition was defined as the functional process that consists of the following four steps: recognition and integration of external information, action according to the integrated information, and memory of that experience. The biological definition of cognition without subjective elements was intended to invite people in many areas into the discussion. According to this definition, the essential elements in immunity superimpose with those of the nervous system. Thus, these two systems are not only functionally related but also similar in a fundamental way, and immunity is identified as cognition. As discussed in the previous sections, the findings in plant immunity are no exception and further strengthen the concept of immunity as cognition. It is needless to say that although they are sometimes used interchangeably, recognition (of pathogens, for example) is only a part of cognition.

Anne-Marie Moulin saw three paradigms in immunity: defensive, selective, and cognitive [103]. The cognitive paradigm, in this case, refers to the recognition of pathogens and functional relatedness with the nervous system but not to the essential similarity between the two systems, as proposed in this essay. Irun Cohen also proposed his version of the cognitive paradigm. He views the immune system as an information handling system that is comparable to the central nervous system. What a cognitive system does is extract information and transform it, by using already deposited information or experience, into an effective means to deal with the external world. A cognitive system has to “know what it should be looking for”, and to be equipped with internal information for dealing with the external milieu. In a way, Cohen sees a sense of direction or a kind of intentionality, but not consciousness in lymphocytes or the immune system [89]. His recent effort was to reframe our view of the immune system in computational terms [91]. The present essay expands previous proposals on the cognitive nature of immunity [89,103,104].

## 4. Conclusions

In this essay, the current scientific understanding of immune phenomena in plants was first reviewed with special reference to biologically defined cognitive activities that contain the following four elements: a recognition of pathogens and pathogen-induced conditions, integration of perceived information, reaction according to integrated information, and memory of that experience [76]. Although the fundamental functional elements have also been conserved in plants, each process exhibited unique features, including pathogen recognition, autoimmunity as a form of disturbed recognition, and memory formation sometimes of transgenerational nature. These characteristics of immune functional elements are superimposed with those of cognitive function exerted by the nervous system. Thus, it is possible that, in principle, immunity includes cognitive functions, although they are not necessarily identical to that of human capacity. A metaphysical analysis, based on these scientific analyses, also points to a possibility that immunity has a conative activity in its essential part, which, according to the Spinozist concept, includes mental elements in addition to physical elements. Given the diversity of living organisms and the understanding of immunity in terms of scientific and metaphysical perspectives, plants are well-positioned in the evolutionary tree to assess how they uniquely perceive and interact with internal and external cues to maintain their integrity and to understand in broader terms what those actions imply and how they help clarify immune functions in other organisms.

## References

[1] Cooper M.D., Alder M.N. (2006). The evolution of adaptive immune systems. Cell.

[2] Litman G.W., Cannon J.P., Dishaw L.J. (2005). Reconstruction immune phylogeny: New perspective. Nat. Rev. Immunol..

[3] Boehm T., McCurley N., Sutoh Y., Schorpp M., Kasahara M., Cooper M.D. (2012). VLR-based adaptive immunity. Annu. Rev. Immunol..

[4] Chisholm S.T., Coaker G., Day B., Staskawicz B.J. (2006). Host-microbe interactions: Shaping the evolution of the plant immune response. Cell.

[5] Conrath U., Beckers G.J.M., Langenbach C.J.G., Jaskiewicz M.R. (2015). Priming for enhanced defense. Annu. Rev. Phytopathol..

[6] Reimer-Michalski E.M., Conrath U. (2016). Innate immune memory in plants. Semin. Immunol..

[7] Spoel S.H., Dong X. (2012). How do plants achieve immunity? Defence without specialized immune cells. Nat. Rev. Immunol..

[8] Kourelis J., van der Hoorn R.A.L. (2018). Defended to the Nines: 25 years of resistance gene cloning identifies nine mechanisms for R protein function. Plant Cell.

[9] Sharrock J., Sun J.C. (2020). Innate immunological memory: From plants to animals. Curr. Opin. Immunol..

[10] Yakura H. Should there be a place for metaphysics in science?. Proceedings of the Fifth Congress of the Society of Philosophy of Sciences.

[11] Yakura H. (2016). Epistemological and Metaphysical Problems Posed by Immunology. Ph.D. Thesis.

[12] Malinovsky F.G., Fangel J.U., Willats W.G.T. (2014). The role of the cell wall in plant immunity. Front. Plant Sci..

[13] Otulak-Kozieł K., Kozieł E., Lockhart B.E.L., Bujarski J.J. (2020). The expression of potato expansin A3 (*StEXPA3*) and extensin4 (*StEXT4*) genes with distribution of StEXPAs and HRGPs-extensin changes as an effect of cell wall rebuilding in two types of PVY^NTN^-*Solanum tuberosum* interactions. Viruses.

[14] Underwood W. (2012). The plant cell wall: A dynamic barrier against pathogen invasion. Front. Plant Sci..

[15] Ding S.W., Voinnet O. (2007). Antiviral immunity directed by small RNAs. Cell.

[16] Voinnet O. (2001). RNA silencing as a plant immune system against viruses. Trends Genet..

[17] Janeway C.A., Medzhitov R. (2002). Innate immune recognition. Annu. Rev. Immunol..

[18] Jones J.D.G., Dangl J.L. (2006). The plant immune system. Nature.

[19] Newman M.A., Sundelin T., Nielsenand J.T., Erbs G. (2013). MAMP (microbe-associated molecular pattern) triggered immunity in plants. Front. Plant Sci..

[20] Liu X., Grabherr H.M., Willmann R., Kolb D., Brunner F., Bertsche U., Kühner D., Franz-Wachtel M., Amin B., Felix G. (2014). Host-induced bacterial cell wall decomposition mediates pattern-triggered immunity in Arabidopsis. eLife.

[21] Zipfel C. (2014). Plant pattern-recognition receptors. Trends Immunol..

[22] Nürnberger T., Brunner F., Kemmering F., Piater L. (2004). Innate immunity in plants and animals: Striking similarities and obvious differences. Immunol. Rev..

[23] Dangl J.L., Horvath D.M., Staskawicz B.J. (2013). Pivoting the plant immune system from dissection to deployment. Science.

[24] Dangl J.L., Jones J.D.G. (2001). Plant pathogens and integrated defence responses to infection. Nature.

[25] Bent A.F. (1996). Plant disease resistance genes: Function meets structure. Plant Cell.

[26] Ellis J., Dodds P., Pryor T. (2000). Structure, function and evolution of plant disease resistance genes. Curr. Opin. Plant Biol..

[27] Ross A.F. (1961). Systemic acquired resistance induced by localized virus infections in plants. Virology.

[28] Biffen R.H. (1907). Studies on the inheritance of disease resistance. J. Agric. Sci..

[29] Flor H.H. (1947). Inheritance of reaction to rust in flax. J. Agric. Res..

[30] Flor H.H. (1955). Host-parasite interaction in flax rust–its genetics and other implications. Phytopathology.

[31] Flor H.H. (1942). Inheritance of pathogenicity in *Melampsora lini*. Phytopathology.

[32] Flor H.H. (1956). The complementary genic systems in flax and flax rust. Adv. Genet..

[33] Flor H.H. (1971). Current status of the gene-for-gene concept. Annu. Rev. Phytopathol..

[34] Dodds P.N., Lawrence G.J., Catanzariti A.-M., Teh T., Wang C.-I.A., Ayliffe M.A., Kobe B., Ellis J.G. (2006). Direct protein interaction underlies gene-for-gene specificity and coevolution of the flax resistance genes and flax rust avirulence genes. Proc. Natl. Acad. Sci. USA.

[35] Deslandes L., Olivier J., Peeters N., Feng D.X., Khounlotham M., Boucher C., Somssich I., Genin S., Marco Y. (2003). Physical interaction between RRS1-R, a protein conferring resistance to bacterial wilt, and PopP2, a type III effector targeted to the plant nucleus. Proc. Natl. Acad. Sci. USA.

[36] Jia Y., McAdams S.A., Bryan G.T., Hershey H.P., Valent B. (2000). Direct interaction of resistance gene and avirulence gene products confers rice blast resistance. EMBO J..

[37] Meyers B.B., Kozik A., Griego A., Kuang H., Michelmore R.W. (2003). Genome-wide analysis of NBS-LRR–encoding genes in Arabidopsis. Plant Cell.

[38] Goff S.A., Ricke D., Lan T.H., Presting G., Wang R., Dunn M., Glazebrook J., Sessions A., Oeller P., Varma H. (2002). A draft sequence of the rice genome (*Oryza sativa* L. ssp. *japonica*). Science.

[39] Mackey D., Holt III B.F., Wiig A., Dangl J.L. (2002). RIN4 interacts with *Pseudomonas syringae* Type III effector molecules and is required for RPM1-mediated resistance in *Arabidopsis*. Cell.

[40] Kim M.G., da Cunha L., McFall A.J., Belkhadir Y., DebRoy S., Dangl J.L., Mackey D. (2005). Two *Pseudomonas syringae* Type III effectors inhibit RIN4-regulated basal defense in *Arabidopsis*. Cell.

[41] Chung E.W., da Cunha L., Wu A.J., Gao Z., Cherkis K., Afzal A.J., Mackey D., Dangl J.L. (2011). Specific threonine phosphorylation of a host target by two unrelated type III effectors activates a host innate immune receptor in plants. Cell Host Microbe.

[42] Liu J., Elmore J.M., Lin Z.J.D., Coaker G. (2011). A receptor-like cytoplasmic kinase phosphorylates the host target RIN4, leading to the activation of a plant innate immune receptor. Cell Host Microbe.

[43] Mackey D., Belkhadir Y., Alonso J.M., Ecker J.R., Dangl J.L. (2003). *Arabidopsis* RIN4 Is a target of the type III virulence effector AvrRpt2 and modulates RPS2-mediated resistance. Cell.

[44] Axtell M.J., Staskawicz B.J. (2003). Initiation of RPS2-specified disease resistance in *Arabidopsis* is coupled to the AvrRpt2-directed elimination of RIN4. Cell.

[45] Matzinger P. (1994). Tolerance, danger, and the extended family. Annu. Rev. Immunol..

[46] Seong S.-Y., Matzinger P. (2004). Hydrophobicity: An ancient damage-associated molecular pattern that initiates innate immune responses. Nat. Rev. Immunol..

[47] Duxbury Z., Ma Y., Furzer O.J., Huh S.U., Cevik V., Jones J.D.G., Sarris P.F. (2016). Pathogen perception by NLRs in plants and animals: Parallel worlds. Bioessays.

[48] Ting J.P.Y., Willingham S.B., Bergstralh D.T. (2008). NLRs at the intersection of cell death and immunity. Nat. Rev. Immunol..

[49] Hatsugai N., Iwasaki S., Tamura K., Kondo M., Fuji K., Ogasawara K., Nishimura M., Hara-Nishimura I. (2009). A novel membrane fusion-mediated plant immunity against bacterial pathogens. Genes Dev..

[50] Coll N.S., Vercammen D., Smidler A., Clover C., Van Breusegem F., Dangl J.L., Epple P. (2010). Arabidopsis type I metacaspases control cell death. Science.

[51] Yu I.C., Fengler K.A., Clough S.J., Bent A.F. (2000). Identification of Arabidopsis mutants exhibiting an altered hypersensitive response in gene-for-gene disease resistance. Mol. Plant Microbe In..

[52] Yu I.C., Parker J., Bent A.F. (1998). Gene-for-gene disease resistance without the hypersensitive response in *Arabidopsis dnd1* mutant. Proc. Natl. Acad. Sci. USA.

[53] Gaffney T., Friedrich L., Vernooij B., Negrotto D., Nye G., Uknes S., Ward E., Kessmann H., Ryals J. (1993). Requirement of salicylic acid for the induction of systemic acquired resistance. Science.

[54] Yalpani N., Silverman P., Wilson T.M., Kleier D.A., Raskin I. (1991). Salicylic acid is a systemic signal and an inducer of pathogenesis-related proteins in virus-infected tobacco. Plant Cell.

[55] Park S.W., Kaimoyo E., Kumar D., Mosher S., Klessig D.F. (2007). Methyl salicylate is a critical mobile signal for plant systemic acquired resistance. Science.

[56] Truman W., Bennett M.H., Kubigsteltig I., Turnbull C., Grant M. (2007). *Arabidopsis* systemic immunity uses conserved defense signaling pathways and is mediated by jasmonates. Proc. Natl. Acad. Sci. USA.

[57] Chanda B., Xia Y., Mandal M.K., Yu K., Sekine K., Gao Q., Selote D., Hu Y., Stromberg A., Navarre D. (2011). Glycerol-3-phosphate is a critical mobile inducer of systemic immunity in plants. Nat. Genet..

[58] Jung H.W., Tschaplinski T.J., Wang L., Glazebrook J., Greenberg J.T. (2009). Priming in systemic plant immunity. Science.

[59] Bomblies K., Weigel D. (2007). Hybrid necrosis: Autoimmunity as a potential gene-flow barrier in plant species. Nat. Rev. Genet..

[60] Bomblies K., Lempe J., Epple P., Warthmann N., Lanz C., Dangl J.L., Weigel D. (2007). Autoimmune response as a mechanism for a Dobzhansky-Muller-type incompatibility syndrome in plants. PLoS Biol..

[61] Tran D.T.N., Chung E.H., Habring-Müller A., Demar M., Schwab R., Dangl J.L., Weigel D., Chae E. (2017). Activation of a plant NLR complex through heteromeric association with an autoimmune risk variant of another NLR. Curr. Biol..

[62] Jeuken M.J., Zhang N.W., McHale L.K., Pelgrom K., den Boer E., Lindhout P., Michelmore R.W., Visser R.G., Niks R.E. (2009). *Rin4* causes hybrid necrosis and race-specific resistance in an interspecific lettuce hybrid. Plant Cell.

[63] Alcázar R., García A.V., Kronholm I., de Meaux J., Koornneef M., Parker J.E., Reymond M. (2010). Natural variation at Strubbelig Receptor Kinase 3 drives immune-triggered incompatibilities between *Arabidopsis thaliana* accessions. Nat. Genet..

[64] Oldroyd G.E.D., Staskawicz B.J. (1998). Genetically engineered broad-spectrum disease resistance in tomato. Proc. Natl. Acad. Sci. USA.

[65] Shirano Y., Kachroo P., Shah J., Klessig D.F. (2002). A gain-of-function mutation in an Arabidopsis Toll Interleukin1 receptor-nucleotide binding site-leucine-rich repeat type R gene triggers defense responses and results in enhanced disease resistance. Plant Cell.

[66] Stokes T.L., Kunkel B.N., Richards E.J. (2002). Epigenetic variation in *Arabidopsis* disease resistance. Genes Dev..

[67] Kim S.H., Gao F., Bhattacharjee S., Adiasor J.A., Nam J.C., Gassmann W. (2010). The Arabidopsis resistance-like gene *SNC1* is activated by mutations in *SRFR1* and contributes to resistance to the bacterial effector AvrRps4. PLoS Pathog..

[68] Li Y., Li S., Bi D., Cheng Y.T., Li X., Zhang Y. (2010). SRFR1 negatively regulates plant NB-LRR resistance protein accumulation to prevent autoimmunity. PLoS Pathog..

[69] Cheng Y.T., Li Y., Huang S., Huang Y., Dong X., Zhang Y., Li X. (2011). Stability of plant immune-receptor resistance proteins is controlled by SKP1-CULLIN1-F-box (SCF)-mediated protein degradation. Proc. Natl. Acad. Sci. USA.

[70] Chester K.S. (1933). The problem of acquired physiological immunity in plants. Q. Rev. Biol..

[71] Kuć J. (1982). Induced immunity ot plant disease. BioScience.

[72] Beckers G.J.M., Jaskiewicz M., Liu Y., Underwood W.R., He S.Y., Zhang S., Conrath U. (2009). Mitogen-activated protein kinases 3 and 6 are required for full priming of stress responses in *Arabidopsis thaliana*. Plant Cell.

[73] Cao H., Bowling S.A., Gordon S., Dong X. (1994). Characterization of an Arabidopsis mutant that is nonresponsive to inducers of systemic acquired resistance. Plant Cell.

[74] Dong X. (2004). NPR1, all things considered. Curr. Opin. Plant Biol..

[75] Luna E., Bruce T.J.A., Roberts M.R., Flors V., Ton J. (2012). Next-generation systemic acquired resistance. Plant Physiol..

[76] Yakura H. (2019). A hypothesis: CRISPR-Cas as a minimal cognitive system. Adapt. Behav..

[77] Brouns S.J., Jore M.M., Lundgren M., Westra E.R., Slijkhuis R.J.H., Snijders A.P.L., Dickman M.J., Makarova L.S., Koonin E.V., van der Oost J. (2008). Small CRISPR RNAs guide antiviral defense in prokaryotes. Science.

[78] Marraffini L.A., Sontheimer E.J. (2008). CRISPR interference limits horizontal gene transfer in straphylococci by targeting DNA. Science.

[79] Sorek R., Koonin V., Hugenholtz P. (2008). CRISPR—A widespread system that provides acquired resistance against phages in bacteria and archaea. Nat. Rev. Microbiol..

[80] Spinoza B. (1677). Ethics.

[81] Block N. (1995). On a confusion about a function of consciousness. Behav. Brain Sci..

[82] Edelman G.M. (2003). Naturalizing consciousness: A theoretical framework. Proc. Natl. Acad. Sci. USA.

[83] Ziv Y., Schwartz M. (2008). Orchestrating brain-cell renewal: The role of immune cells in adult neurogenesis in health and disease. Trends Mol. Med..

[84] Veiga-Fernandes H., Artis D. (2018). Neuronal-immune system cross-talk in homeostasis. Science.

[85] Mack M.R., Kim B.S. (2018). The itch-scratch cycle: A neuroimmune perspective. Trends Immunol..

[86] Maier S.F., Watkins L.R. (2000). The immune system as a sensory system: Implications for psychology. Curr. Dir. Psychol. Sci..

[87] Blalock J.E. (2005). The immune system as the sixth sense. J. Intern. Med..

[88] Ader R. (2006). Psychoneuroimmunology.

[89] Cohen I.R. (1992). The cognitive paradigm and the immunological homunculus. Immunol. Today.

[90] Cohen I.R., Young D.B. (1991). Autoimmunity, microbial immunity and the immunological homunculus. Immunol. Today.

[91] Cohen I.R. (2007). Real and artificial immune systems: Computing the state of the body. Nat. Rev. Immunol..

[92] Ehrlich P., Morgenroth J. (1900). Über haemolysine. Dritte Mittheilung. (On hemolysis. Third communication). Berl. Klin. Wochenschr..

[93] Cohen I.R. (2007). Biomarkers, self-antigens and the immunological homunculus. J. Autoimmun..

[94] Stern A., Keren L., Wurtzel O., Amitai G., Sorek R. (2010). Self-targeting by CRISPR: Gene regulation or autoimmunity?. Trends Genet..

[95] Marraffini L.A., Sontheimer E.J. (2010). Self versus non-self discrimination during CRISPR RNA-directed immunity. Nature.

[96] Netea M.G., Domínguez-Andrés J., Barreiro L.B., Chavakis T., Divangahi M., Fuchs E., Joosten L.A.B., van der Meer J.W.M., Mhlanga M.M., Mulder W.J.M. (2020). Defining trained immunity and its role in health and disease. Nat. Rev. Immunol..

[97] Pecinka A., Rosa M., Schikora A., Berlinger M., Hirt H., Luschnig C., Scheid O.M. (2009). Transgenerational stress memory is not a general response in Arabidopsis. PLoS ONE.

[98] Molinier J., Ries G., Zipfel C., Hohn B. (2006). Transgeneration memory of stress in plants. Nature.

[99] Slaughter A., Daniel X., Flors V., Luna E., Hohn B., Mauch-Mani B. (2012). Descendants of primed Arabidopsis plants exhibit resistance to biotic stress. Plant Physiol..

[100] Berendsen M.L.T., Øland C.B., Bles P., Jensen A.K.G., Kofoed P.E., Whittle H., de Bree L.C.J., Netea M.G., Martins C., Benn C.S. (2020). Maternal priming: Bacillus Calmette-Guérin (BCG) vaccine scarring in mothers enhances the survival of their child with a BCG vaccine scar. J. Pediatr. Infect. Dis. Soc..

[101] Moore R.S., Kaletsky R., Murphy C.T. (2019). Piwi/PRG-1 argonaute and TGF-β mediate transgenerational learned pathogenic avoidance. Cell.

[102] Fleck L. (1979). The Genesis and Development of a Scientific Fact.

[103] Moulin A.M., Daëron M. (1995). Clés pour l’histoire de l’immunologie (Keys for the history of immunology), In Le Système Immunitaire ou L’immunité Cent ans Après Pasteur (The Immune System or Immunity a 100 Years after Pasteur).

[104] Tauber A.I. (2013). Immunology’s theories of cognition. Hist. Phil. Life Sci..

